# Vim line technique thalamotomy for Parkinson tremor: Case series

**DOI:** 10.1016/j.ijscr.2020.11.096

**Published:** 2020-11-20

**Authors:** Achmad Fahmi, Heri Subianto, Priya Nugraha, Muhammad Hamdan, Asra Al Fauzi, Anggraini Dwi Sensusiati, Budi Utomo, Riyanarto Sarno, Agus Turchan, Mohammad Hasan Macfoed, Takaomi Taira, Abdul Hafid Bajamal

**Affiliations:** a Post Graduate Doctoral Program, Faculty of Medicine, Universitas Airlangga, Surabaya, Indonesia; b Department of Neurosurgery, Faculty of Medicine, Universitas Airlangga, Surabaya, Indonesia; c Department of Neurology, Faculty of Medicine, Universitas Airlangga, Surabaya, Indonesia; d Department of Radiology, Faculty of Medicine, Universitas Airlangga, Surabaya, Indonesia; e Department of Public Health and Preventive Medicine, Faculty of Medicine, Universitas Airlangga, Surabaya, Indonesia; f Department of Informatics, Institut Teknologi Sepuluh November, Surabaya, Indonesia; g Department of Neurosurgery, Tokyo Women’s Medical University Hospital, Tokyo, Japan

**Keywords:** Parkinson tremor, Thalamotomy, Vim line technique

## Abstract

**Introduction:**

The ventral intermediate (Vim) nucleus of the thalamus is difficult to identify even with 3 T magnetic resonance imaging. Stereotactic Vim thalamotomy is a usual procedure to control Parkinson tremor. Successful relieving of the tremor depends on the accuracy of defining the Vim location.

**Presentation of cases:**

Three patients with Parkinson tremor were subjected to stereotactic thalamotomy using the Vim line technique (VLT) so as to precisely determine the Vim location. All patients showed good results, with improved tremors, as indicated by the UPDRS score, without any complications.

**Discussion:**

The precise targeting of the Vim nucleus is crucial importance for the successful Vim thalamotomy. Various method has been developed to determine Vim location. Atlas based and Guiot’s technique routinely used by neurosurgeon. VLT is a new technique that has been developed to determine the Vim location on MRI.

**Conclusion:**

VLT is useful for the determination of the Vim location. However, further research is warranted to prove its effectiveness.

## Highlights

• Vim thalamotomy is a usual procedure to control Parkinson tremor.
• The ventral intermediate nucleus of the thalamus is difficult to identify.
• The precision is crucial importance for the successful Vim thalamotomy.
• Vim line technique (VLT) is useful for the determination of the vim location.

## 1 Introduction

Ventral intermediate (Vim) nucleus of the thalamus is reputed to be ‘invisible’ on the routinely available imaging techniques. Currently, there exists several hypotheses related to the estimation of the Vim location [6]. The use of magnetic resonance imaging (MRI) to identify critical structures near the stereotactic targets is crucial. The optic tract, internal segment of the globus pallidus, border between the Vim nucleus of the thalamus and the internal capsule must be evaluated precisely before some serious surgeries [5].

Several different surgical procedures have been proposed for the treatment of essential tremor, including deep brain stimulation (DBS), radiofrequency thalamotomy, gamma knife radio surgical thalamotomy and focused ultrasound thalamotomy [1]. Stereotactic lesioning surgeries such as thalamotomy and pallidotomy have been performed to control movement disorders. The thalamic Vim nucleus is a usual target for controlling the tremor. Tremor-dominant hemi-Parkinson’s disease (PD) is a good indication of Vim thalamotomy, especially when tremor is a major complaint [5]. Some techniques have been developed to define the Vim location. For instance, the Guiot’s technique uses 3/12 or 4/12 anterior from posterior commissure (PC) in anterior commissure-PC (AC-PC) line [6]. The coordinate base uses the brain atlas to define the Vim location [2]. New techniques are hence required to develop variation of the brain anatomy. Improvement after Vim thalamotomy in the control of tremor ranges between 74% and 90% [1]. VLT did not interfered by this variation. VLT can be used by neurosurgeons to determined the Vim location, including in different anatomical variation of the brain. Using VLT to determine Vim location should increase the outcome that can be measured by Unified PD Rating Scale (UPDRS) score. However, there is insufficient evidence to support or refute efficacy superiority of DBS or thalamotomy for treating essential tremor [7]. All of the procedures performed by the authors in this study involving human participants were in accordance with the ethical standards of the institutional research committee. This work has been reported in line with the PROCESS guideline [8]. This work has been registered at http://www.researchregistry.com (researchregistry6183).

### 1.1 Presentation of cases

#### 1.1.1 Case 1

A 30-year-old man presented with tremor in his right hand and a stiffness at his right side of the body. He was diagnosed with PD since the past 5 years and was receiving oral medication (L-dopa, trihexyphenidyl, pramipexole and ropinirole), but the tremor continued to recur.

**Fig. 1:**
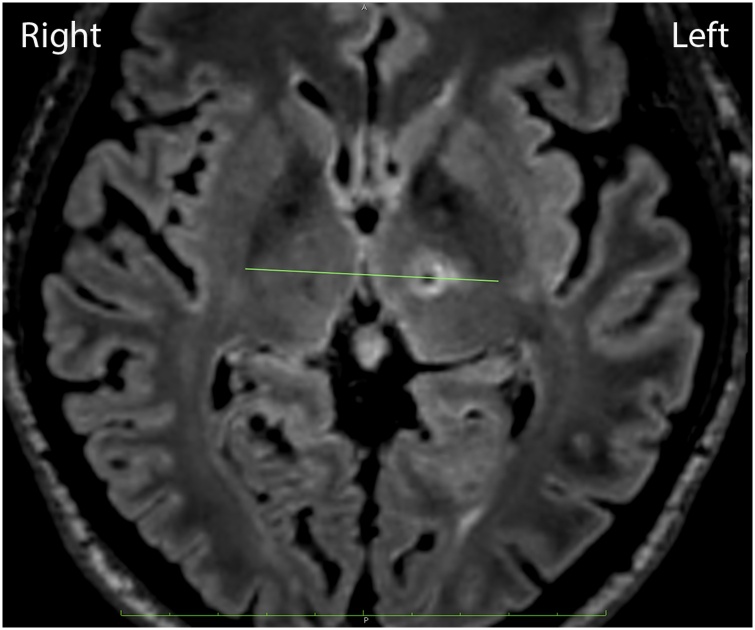
Left side thalamotomy in the Vim line connecting the posterior tips of both the external globus pallidus in the AC-PC plane, 1.5-mm medial from the cerebrospinal tract border.

The MRI and computerized tomography (CT) of the head is performed before conducting stereotactic surgery. The patient’s UPDRS before surgery was ON 6 and OFF 33. The patient consented to undergo left Vim thalamotomy by the Vim line technique (VLT) using the *Cosman* G4 radiofrequency system (electrode active tip 4 mm, diameter 1.1 mm, 70 °C for 30 s) (Fig. 1). Evaluation after surgery revealed that tremor in the right hand and the stiffness of the right side of the body had improved. The patient’s UPDRS score after surgery was ON 0 and OFF 7.

#### 1.1.2 Case 2

A 59-year-old woman presented with a tremor at her left and right sides of the body. The left side tremor was dominant since past 15 years and the right side tremor since past 3 years. She also complained of rigidity and bradykinesia. Patient had ON-OFF response to oral medications (L-dopa, trihexyphenidyl, pramipexole and ropinirole).

**Fig. 2:**
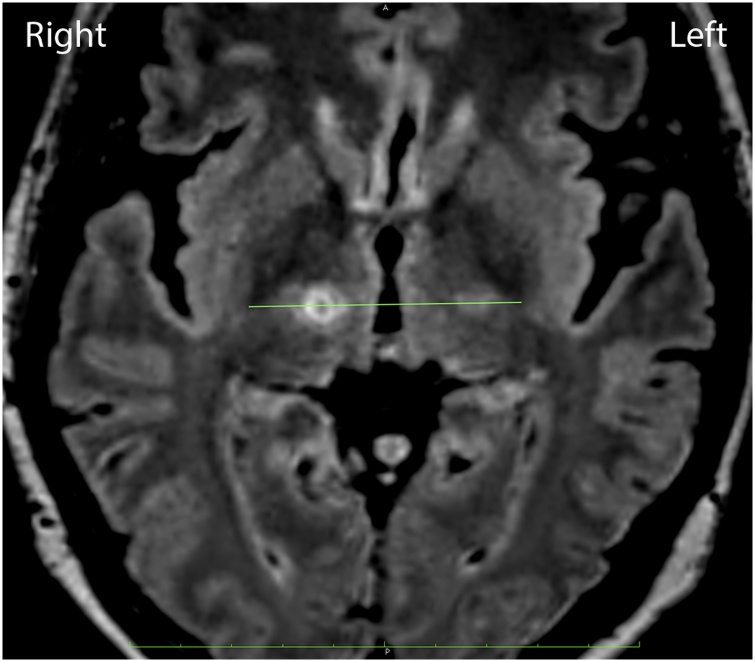
Right-side thalamotomy in the Vim line connecting the posterior tips of both the external globus pallidus in the AC-PC plane, 1.5-mm medial from the cerebrospinal tract border.

The patient’s head MRI and CT scan were performed before the stereotactic surgery. Her UPDRS score before the surgery was ON 15 and OFF 41. The patient consented to undergo the right Vim thalamotomy with VLT using the *Cosman* G4 radiofrequency system (electrode active tip 4 mm, diameter 1.1 mm, 70 °C for 30 s) (Fig. 2). Her evaluation after the surgery revealed that the tremor in the left side of the body had improved. Her UPDRS score after the surgery was ON 0 and OFF 7.

#### 1.1.3 Case 3

**Fig. 3:**
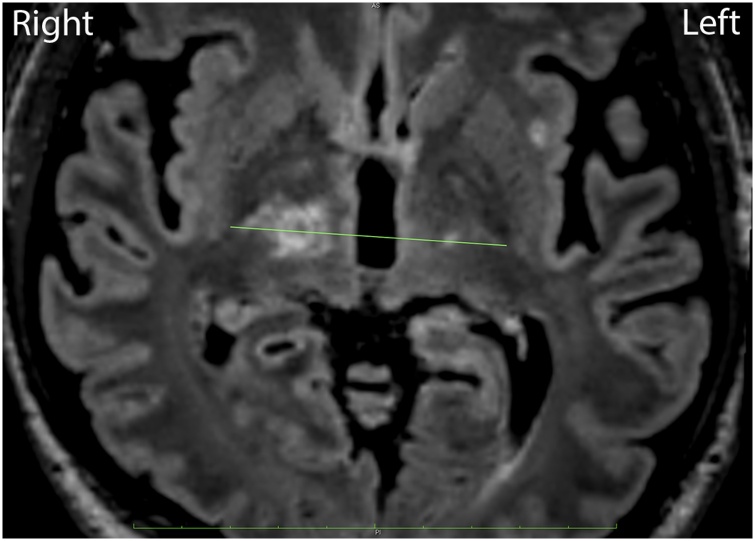
Left-side thalamotomy in the Vim line connecting the posterior tips of both the external globus pallidus in the AC-PC plane, 1.5-mm medial from the cerebrospinal tract border.

A 57-year-old man presented with tremor in the left hand since the past 2 years. He had undergone left Vim thalamotomy 4 years ago for his right-side tremor. His head MRI and CT scan were performed before the stereotactic surgery. His UPDRS score before the surgery was ON 13 and OFF 42 with oral medications (L-dopa, trihexyphenidyl, pramipexole and ropinirole). He consented to undergo right Vim thalamotomy with VLT using the *Cosman* G4 radiofrequency system (electrode active tip 4 mm, diameter 1.1 mm, 70 °C for 30 s) (Fig. 3). His evaluation after the surgery revealed that the tremor in the left hand had improved. His UPDRS score after the surgery was ON 4 and OFF 23.

## 2 Discussion

Thalamic stimulation and thalamotomy are equally effective in reducing drug resistant tremor, and stimulation has only a few adverse effects. The efficacy of stimulation ranges from 71% to 94% in PD patients and from 74% to 90% for essential tremor [1,4]. In patients with bilateral tremor, thalamic stimulation in the dominant hemisphere can be combined with thalamotomy for the non-dominant hemisphere; however, morbidity associated with thalamotomy probably outweighs any advantage of this approach [3].

The precise targeting of the Vim nucleus is crucial importance for the successful Vim thalamotomy. Targeting is usually conducted by using stereotactic coordinates that are specified in relation to a point on the AC-PC line based on the brain atlas or intraoperative stimulation techniques, although these methods sometimes need modification based on the anatomical brain variation. Since the identification of the thalamic nuclei on conventional imaging modalities is difficult, recently, MRI tractography was successfully used to investigate the dentatorubrothalamic tract that crosses the Vim nucleus [2]. Some of the stereotactic planning software available presently do not have the tractography facility.

VLT is a new technique that has been developed to determine the Vim location on MRI. This technique suitable for any brain anatomical variation. This technique is performed by drawing a line that connects both the external globus palidus posterior tips in the AC-PC plane. The Vim location is defined in 1.5-mm medial area from the cerebrospinal tract area border. Vim thalamotomy done with VLT using the *Cosman* G4 radiofrequency system (electrode active tip 4 mm, diameter 1.1 mm, 70 °C for 30 s).

A past study has reported improvement in the UPDRS scores in the long-term evaluation after Vim thalamotomy [4]. We noted symptom improvement in our cases as depicted by their UPDRS scores. Sixth months follow up of UPDRS were examined by same neurologist at the same hospital with constant score.

## 3 Conclusion

Our results depict that stereotactic thalamotomy using VLT can be used to determine the Vim location as a useful approach to reduce or control tremor in PD patients. However, further research is warranted to prove the effectiveness of the proposed approach.

## Declaration of Competing Interest

The authors report no declarations of interest.

## Sources of funding

None.

## Ethical approval

This research had ethical clearance from Dr. Soetomo General Academic Hospital Ethical Committee (No.1619/KEPK/XI/2019).

## Consent

Written informed consent was obtained from the patient for publication of this case report and accompanying images. A copy of the written consent is available for review by the Editor-in-Chief of this journal on request.

## Author contribution

Achmad Fahmi, MD, PhD: study concept or design, data collection, writing the paper.

Heri Subianto, MD: data collection.

Priya Nugraha, MD: data analysis.

Muhammad Hamdan, MD, PhD: Supervising.

Asra Al Fauzi MD, PhD: study concept.

Anggraini Dwi Sensusiati MD, PhD: critical revised article.

Budi Utomo MD, PhD: data analysis or interpretation.

Prof. Riyanarto Sarno: critical revised article.

Prof Takaomi Taira: supervising.

Agus Turchan MD, PhD: supervising.

Prof. Mohammad Hasan Macfoed: supervising.

Prof. Abdul Hafid Bajamal: supervising.

## Registration of research studies

1. Name of the registry: http://www.researchregistry.com
2. Unique identifying number or registration ID: researchregistry6183
3. Hyperlink to your specific registration (must be publicly accessible and will be checked): n/a

## Guarantor

Achmad Fahmi, MD, Ph.D.

Post Graduate Doctoral Program, Faculty of Medicine, Universitas Airlangga, Indonesia.

Agus Turchan, MD, PhD.

Head of Neurosurgery Department, Faculty of Medicine, Universitas Airlangga, Indonesia.

## Provenance and peer-reviewed

Not commissioned, externally peer-reviewed.

## References

[1] Dallapiazza R.F., Lee D.J., De Vloo P., Fomenko A., Hamani C., Hodaie M., Kalia S.K., Fasano A., Lozano A.M. (2019). Outcomes from stereotactic surgery for essential tremor. J. Neurol. Neurosurg. Psychiatry.

[2] Kincses Z.T., Szabó N., Valálik I., Kopniczky Z., Dézsi L., Klivényi P., Jenkinson M., Király A., Babos M., Vörös E., Barzó P., Vécsei L. (2012). Target identification for stereotactic thalamotomy using diffusion tractography. PLoS One.

[3] Schuurman P.R., Bosch D.A., Bossuyt P.M., Bonsel G.J., van Someren E.J., de Bie R.M., Merkus M.P., Speelman J.D. (2000). A comparison of continuous thalamic stimulation and thalamotomy for suppression of severe tremor. N. Engl. J. Med..

[4] Schuurman P.R., Bosch D.A., Merkus M.P., Speelman J.D. (2008). Long-term follow-up of thalamic stimulation versus thalamotomy for tremor suppression. Mov. Disord..

[5] Taira T., Horisawa S., Takeda N., Ghate P. (2018). Stereotactic radiofrequency lesioning for movement disorders. Prog. Neurol. Surg..

[6] Vassal F., Coste J., Derost P., Mendes V., Gabrillargues J., Nuti C., Durif F., Lemaire J.J. (2012). Direct stereotactic targeting of the ventrointermediate nucleus of the thalamus based on anatomic 1.5-T MRI mapping with a white matter attenuated inversion recovery (WAIR) sequence. Brain Stimul..

[7] Zesiewicz T.A., Elble R.J., Louis E.D., Gronseth G.S., Ondo W.G., Dewey R.B., Okun M.S., Sullivan K.L., Weiner W.J. (2011). Evidence-based guideline update: treatment of essential tremor: report of the Quality Standards Subcommittee of the American Academy of Neurology. Neurology.

[8] Agha R.A., Borrelli M.R., Farwana R., Koshy K., Fowler A.J., Orgill D.P., SCARE Group (2018). The PROCESS 2018 statement: updating consensus Preferred Reporting Of CasE Series in Surgery (PROCESS) guidelines. Int. J. Surg..

